# Low Intensity Shockwave Treatment Modulates Macrophage Functions Beneficial to Healing Chronic Wounds

**DOI:** 10.3390/ijms22157844

**Published:** 2021-07-22

**Authors:** Jason S. Holsapple, Ben Cooper, Susan H. Berry, Aleksandra Staniszewska, Bruce M. Dickson, Julie A. Taylor, Paul Bachoo, Heather M. Wilson

**Affiliations:** 1 School of Medicine, Medical Sciences & Dentistry, Institute of Medical Sciences, University of Aberdeen, Foresterhill Road, Aberdeen AB25 2ZD, UK; rz3ro@hotmail.com (J.S.H.); akstaniszewska@gmail.com (A.S.); s.h.berry@abdn.ac.uk (S.H.B.); bruce123dickson@outlook.com (B.M.D.); j.a.taylor@abdn.ac.uk (J.A.T.); 2 Department of Vascular Surgery, NHS Grampian, Foresterhill Road, Aberdeen AB25 2ZN, UK; ben.cooper2@nhs.scot (B.C.); paul.bachoo@nhs.scot (P.B.)

**Keywords:** macrophage, chronic wounds, healing, shockwave therapy, phagocytosis, cytokine, inflammation

## Abstract

Extracorporeal Shock Wave Therapy (ESWT) is used clinically in various disorders including chronic wounds for its pro-angiogenic, proliferative, and anti-inflammatory effects. However, the underlying cellular and molecular mechanisms driving therapeutic effects are not well characterized. Macrophages play a key role in all aspects of healing and their dysfunction results in failure to resolve chronic wounds. We investigated the role of ESWT on macrophage activity in chronic wound punch biopsies from patients with non-healing venous ulcers prior to, and two weeks post-ESWT, and in macrophage cultures treated with clinical shockwave intensities (150–500 impulses, 5 Hz, 0.1 mJ/mm^2^). Using wound area measurements and histological/immunohistochemical analysis of wound biopsies, we show ESWT enhanced healing of chronic ulcers associated with improved wound angiogenesis (CD31 staining), significantly decreased CD68-positive macrophages per biopsy area and generally increased macrophage activation. Shockwave treatment of macrophages in culture significantly boosted uptake of apoptotic cells, healing-associated cytokine and growth factor gene expressions and modulated macrophage morphology suggestive of macrophage activation, all of which contribute to wound resolution. Macrophage ERK activity was enhanced, suggesting one mechanotransduction pathway driving events. Collectively, these in vitro and in vivo findings reveal shockwaves as important regulators of macrophage functions linked with wound healing. This immunomodulation represents an underappreciated role of clinically applied shockwaves, which could be exploited for other macrophage-mediated disorders.

## 1. Introduction

Chronic non-healing wounds of the skin, such as venous or diabetic foot ulcers, are globally a major cause of morbidity and burden to health services, the incidence of which is rising due to the increase in co-morbidities such as diabetes and vascular diseases [1]. They have a complex etiology and closure continues to be challenging with current guideline treatments that include compression or pressure offloading, debridement, infection control, and local ulcer care with various multilayer wound dressings [2,3]. These treatments are not effective in a large percentage of patients and relapse occurs, thus additional advanced care therapeutic approaches are required to induce healing or shorten healing time.

Extracorporeal shockwave therapy (ESWT), has been used clinically for several conditions including urological lithotripsy [4], scars, tendonitis, non-union fractures, plantar fasciitis and osteonecrosis, with few side effects [5,6,7,8]. Potential for such a therapy, using comparatively low shockwave intensities, has been recognized clinically for diabetic and venous ulcer management and is a feasible non-invasive method for improving chronic wound healing [9,10]. ESWT also improves skin flap survival, burn wound healing, and blood flow perfusion in animal studies [11]. Much of the effects induced by ESWT relate to induced neovascularization that improves blood flow perfusion and enhanced cell proliferation to accelerate tissue regeneration [12]. It has also been suggested that ESWT dampens the release of pro-inflammatory mediators to improve healing in experimental models [13]; however, the full molecular and cellular effects have not been characterized.

Chronic wounds are characterized by persistent inflammation. Macrophages are heterogeneous immune cells that play key roles in inflammation and all stages of the wound healing process, as well as in host defense responses and restoring and maintaining homeostasis following challenges from injury, infection, or malignancy [14]. Macrophages facilitate healing directly by promotion and resolution of inflammation and phagocytosis of cell debris, apoptotic cells, and microbes [15]. They secrete a variety of factors, including cytokines, which support angiogenesis, extracellular matrix synthesis, fibroblast proliferation, and epithelialization, therefore facilitating regeneration of the injured tissue. In chronic non-healing wounds, there is a stalling of the inflammatory phase with excess proinflammatory macrophages and consequently an inability to resolve the wound [16]. Targeting and correcting cellular and molecular causes of prolonged and unrestrained inflammation in chronic wounds represents a potential therapeutic strategy to return them to a healing state and restart wound resolution.

The phenotype and functions that macrophages develop are determined by integration of multiple signals from their microenvironment [14]. In vitro, microbial stimuli, for example, LPS (M1 activation), can drive the proinflammatory, tissue-destructive properties of macrophages, whereas IL-4, IL-10 or uptake of apoptotic cells (M2 activation) induces polarization to a more tissue-reparative phenotype [14]. This M1 and M2 macrophage classification, based on in vitro activation studies, however, does not accurately represent macrophage function in vivo, where there is a continuum of phenotypes due to the complexity of the activating environment and where the cells modify their role based on the needs of the tissue [17]. The microenvironmental stimuli driving the different macrophage phenotypes and functions have been predominantly characterized as biologic and chemical stimuli, such as infectious products, cytokines, and metabolic factors. However, recent studies have shown macrophages are mechanosensitive and can also respond to physical stimuli e.g., stretch, matrix topography and electrical stimulation [18,19].

In light of the clinical evidence that ESWT promotes resolution of inflammatory processes and tissue repair and the key role of macrophages in these events, the purpose of the work was to determine how low intensity shockwave stimulation influences the response and functional properties of macrophages, both in patient biopsies and in cells in vitro, and to identify potential underlying mechanisms of any responses generated. We provide a new insight into the role of shockwaves as important contributors to the co-ordination and regulation of macrophage wound resolution functions that could have important clinical implications for other macrophage-mediated disorders.

## 2. Results

### 2.1. ESWT Enhances Healing of Chronic Ulcers

**Figure 1:**
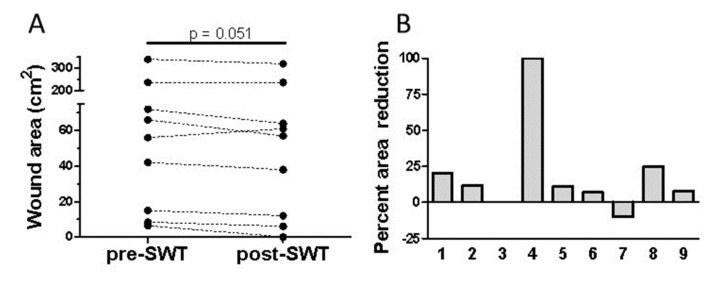
Extracorporeal shock wave therapy (ESWT) improves healing of chronic ulcers. (**A**) ESWT improved healing in seven out of the nine patients as determined by a decrease in total wound area (cm^2^) from baseline before ESWT and at 2 weeks post-therapy. (**B**) Seven out of nine patients showed a decrease in the percentage area of their wound from baseline to 2 weeks post-ESWT, one patient showed no change and one patient a decrease in reduction. Numbers shown on the x-axis (1–9) represent individual patients.

Ten patients, five males and five females, were recruited to the study (age range 36–82 years old). These patients had venous ulcers that had not shown any signs of healing for ≥8 weeks. One patient had only a pre-ESWT biopsy taken, as at follow up had medical complications and was excluded from the analysis. Comparing biopsies from the same patient before and after ESWT was important in this small-scale study to reduce the between-subject variance in responses that can occur. The base area of wounds ranged in size from 6.4–340 cm^2^. Of the nine patients included in the study, ESWT improved healing in seven, as assessed by a decreased measurement of wound area in the patient at baseline before ESWT, and at two weeks post-shockwave therapy (Figure 1A), although this decrease did not quite reach statistical significance (mean values ± SEM, pre-treatment 93.53 ± 38.61; post-treatment 88.23 ± 37.40; *p* = 0.051). The median reduction in wound area size after one round of ESWT was 11% with an interquartile range of 1.5–22.5%. A decrease in the percentage of wound area before and after ESWT was noted for the majority of patients (Figure 1B). For one patient the wound area increased and in one patient there was no change in wound area from baseline. Therefore, for most patients, one application of ESWT improved healing in wounds that had not shown any change in area size for ≥8 weeks despite conventional treatments. Further rounds of ESWT continued to improve healing for most of these patients as determined by a decrease in the area of wound (data not included).

**Figure 2:**
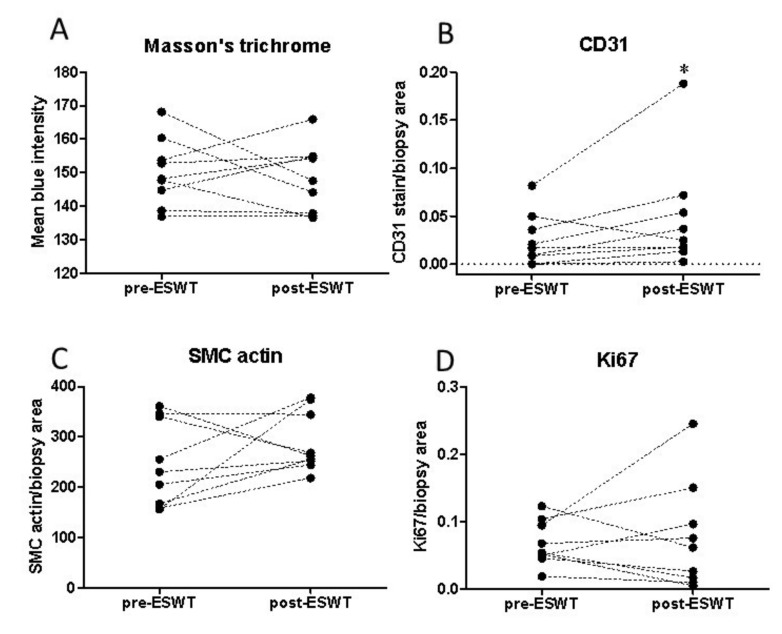
Extracorporeal shock wave therapy (ESWT) improves angiogenic markers in patient wound biopsies. (**A**) Collagen fiber deposition was quantified and presented as mean blue intensity of Masson‘s Trichrome stain in patient biopsies before and two weeks post-ESWT treatment. (**B**) Quantification of the CD31 stained area and (**C**) smooth muscle cell actin (SMC) stained area was determined as a marker of angiogenesis and (**D**) Ki67 staining per total wound biopsy area represented cell proliferation in individual patients pre- and post-treatment. Graphs represent values from individual patients; *n* = 9; * represents differences between the mean values before and after ESWT treatment, *p* < 0.05.

Next, patient biopsies taken before and after ESWT were analyzed morphologically to determine how this related to improvements in healing following ESWT. Wound healing can relate to a change in collagen structure and abundance. To determine whether ESWT affected collagen fiber accumulation, Masson’s trichrome stained sections were analyzed by mean intensity of staining in patient pre- and post-ESWT ulcer biopsies (Figure 2A). There were, however, no significant differences observed in the intensity of staining of collagen fibers per biopsy area after ESWT (Figure 2A; pre-treatment: 151.8 ± 3.3, post-treatment: 149.5 ± 3.5, *p* = 0.618) despite the increased healing observed. This may relate to the short time scale (14 days) for collagen fiber analysis between pre- and post-SWT treatment or remodeling events taking place.

A key role of ESWT previously reported to enhance wound healing in patients is an increase in angiogenesis [12]. To determine potential effects of ESWT on angiogenesis in our study the area of CD31-positive stained vessels relative to the total biopsy area was determined pre- and 14 days post-ESWT. The staining per biopsy area of CD31 increased in the majority of patients (seven out of nine patients) relating to the increased healing in these patients (pre-ESWT 0.025 ± 0.01 vs. post-ESWT 0.047 ± 0.02, *p* = 0.0391); Figure 2B. Smooth muscle cell (SMC) actin levels also relate to vessel abundance. SMC staining per biopsy area of wound increased in six out of nine patients, (mean levels pre- and post-ESWT 246.41 ± 27.93; 288.32 ± 20.04; *p* = 0.085); Figure 2C. These results support the concept that shockwave therapy can enhance angiogenesis in human chronic ulcers. Cell proliferation, important in repairing wounds, was assessed by immunostaining with the proliferation marker Ki67 and stain intensity per biopsy area determined (Figure 2D). Proliferation, as determined by Ki67 staining, increased in 5/9 patients following ESWT treatment (pre-ESWT 0.067 ± 0.011 vs. post-ESWT 0.076 ± 0.026; *p* = 0.398) although the difference pre- and post-ESWT did not reach statistical significance.

### 2.2. ESWT Modulates Macrophage Abundance in Chronic Ulcers

**Figure 3:**
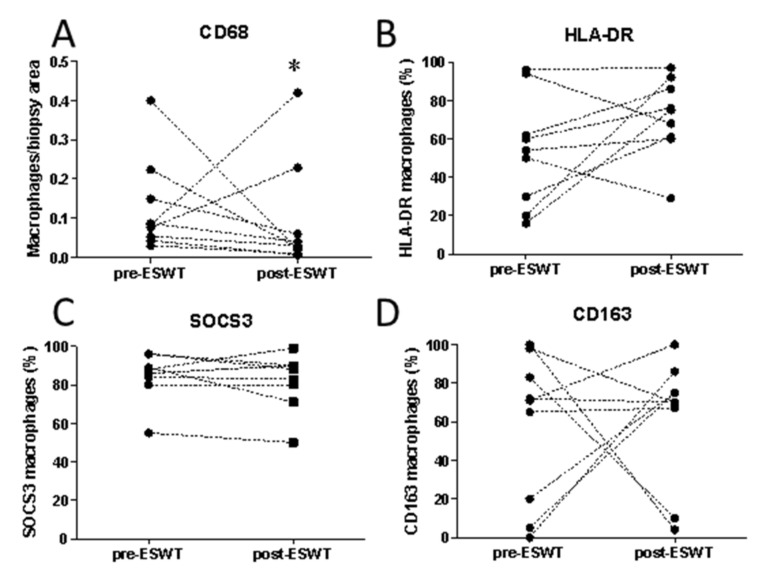
Extracorporeal shock wave therapy (ESWT) alters the number and activation of macrophages in wound biopsies. The number (**A**) and activation status of macrophages (**B**–**D**) was determined by immunohistochemistry in patient biopsies before and after ESWT. Macrophage activation markers were defined as (**B**) HLA DR and (**C**) SOCS3 for M1 activated macrophages and (**D**) CD163 for M2 activated macrophages. * represents differences between values before and after ESWT treatment, *p* < 0.05.

As macrophages are key in the healing process, it was important to determine the effects on macrophage responses in patient biopsies before and after ESWT. There was a significant difference in number of macrophages per biopsy area (Figure 3A) pre- and post-ESWT (0.12 ± 0.036 vs. 0.093 ± 0.047; *p* = 0.048). In all but two patients the macrophage counts per wound area decreased after ESWT, as compared with untreated base level counts (Figure 3A). The two patients that did not show a decrease in the number of macrophages per wound biopsy area were those who did not exhibit an improvement in healing. This suggests a decrease in macrophage number could be an important factor in the facilitated healing induced by ESWT.

We next analyzed the phenotype of these macrophages using double immunohistochemistry of macrophages and defined markers [19]. M1-like macrophages are pro-inflammatory and can lead to tissue injury while M2-like macrophages are involved in healing and anti-inflammatory functions [14]. Of the nine patients analyzed, the percentage of M1-like macrophages as determined by SOCS3 expression decreased in 4 and increased in 4 out of 9 patients, with one showing no change in expression (Figure 3B). For the M1 activation marker HLA DR, 7 out of 9 patients showed an increase in expression (Figure 3C). For the M2 activation marker CD163, percentages increased in 5 of the 9 patients, remained static in one patient and decreased in the remaining 3 (Figure 3D). Thus, two weeks post-shockwave therapy, regardless of the decrease in overall macrophage numbers, generally increased overall activation of macrophages (over 60% increased) in chronic wounds, irrespective of the M1- or M2-like phenotypes. Activation markers were, however, still expressed in those macrophages where no increase was observed.

### 2.3. Shockwave Stimulation Enhances Macrophage Phagocytosis of Apoptotic Cells

**Figure 4:**
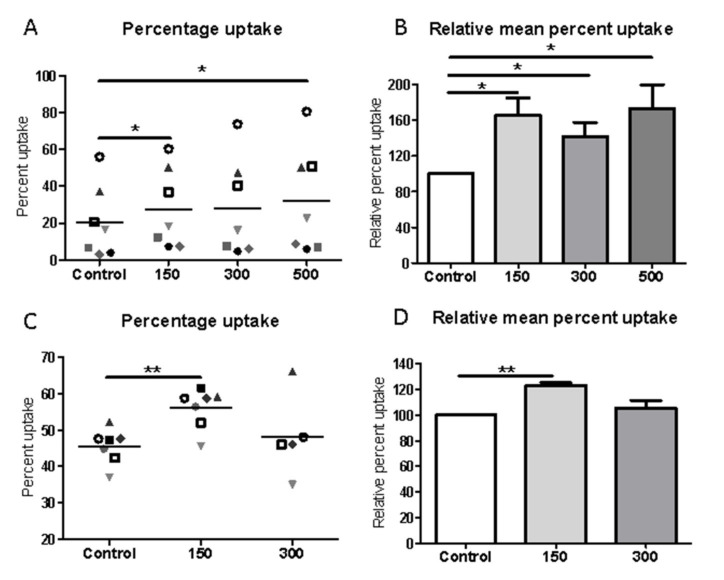
Shockwave stimulation up-regulates macrophage phagocytosis of apoptotic cells. Macrophages were unstimulated (control) or exposed to shockwave intensities of 150–500; 5 Hz, 0.1 mJ/mm^2^ then cultured with apoptotic cells for typically 2 h. (**A**) Percentage uptake and (**B**) relative percentage uptake of apoptotic cells by J774 macrophages. (**C**) Percentage uptake and (**D**) relative percentage uptake of apoptotic cells by human monocyte-derived macrophages. Data plotted represent individual values as shown by each symbol with mean value presented (**A**,**C**) and the normalized data with means ± SEM determining the relative percentage of shock wave exposed cells to control cells for each cell preparation (**B**,**D**). Data from experiments from 5–7 individual macrophage preparations. * represents *p* < 0.05; ** represents < 0.001 compared to unstimulated, control macrophages.

Given that one round of ESWT improved healing in chronic ulcers and a key effect was on macrophage number per biopsy area and activation status, the next experiments addressed mechanistically how shockwaves could potentially influence macrophage healing functions per se, using an in vitro cell culture assay [20]. Macrophages are critical in the clearance of apoptotic cells in wounded tissue and subsequent switching of their function from pro-inflammatory to healing [15]. To address the effect of shockwave on macrophage phagocytosis, the percent uptake of apoptotic cells was calculated with and without exposure to a range of shockwave intensities similar to that administered to patient ulcers (150–500 impulses, 5Hz, 0.1 mJ/mm^2^). Macrophages (J774 macrophage cell line) that had been exposed to shockwaves demonstrated an overall increase in phagocytosis (Figure 4A) compared to uptake by unexposed control cells. The uptake of apoptotic cells in macrophages exposed to 500 impulses of shockwaves rose from 20.5 ± 7.4% to 32.2 ± 10.9%, *p* = 0.037, a mean increase over baseline phagocytosis of 73.2%, (Figure 4B). Macrophage preparations from every experiment tested displayed such increased phagocytic responses. As shown in Figure 4A, increases in the percentage phagocytic uptake were also observed when macrophages were exposed to shockwaves of lower intensities; (control 20.5 ± 7.4% versus 150 impulses group 27.5 ± 8.2%, *p* = 0.0146; increase over baseline of 65.4% and versus 300 impulses group, 28.1 ± 10.0%, *p* = 0.059; increase over baseline of 41.8%). This increase in uptake of apoptotic cells following shockwave treatment was confirmed when primary human blood-derived macrophages were used; control non-shockwave exposed, 45.5 ± 1.8% versus 150 impulse group, 56.0 ± 2.1%, *p* < 0.0001 and 300 impulse group 48.2 ± 5%, *p* = 0.358; Figure 4C. There was also a significant relative percent increase over baseline phagocytosis by 23.1 ± 1.9% in the 150 impulse group; Figure 4D. Strikingly, this demonstrates that shockwave treatment is associated with a significant increase in uptake of apoptotic cells by both J774 and human macrophages at the 150 and 500 impulses or 150 impulses, respectively. These shockwave-induced effects could be predicted to stimulate biologically relevant increases in the clearance of apoptotic cells in physiologic settings.

### 2.4. Shockwave Stimulation Does Not Affect Macrophage Phagocytosis of Beads as a Non-Specific Substrate

**Figure 5:**
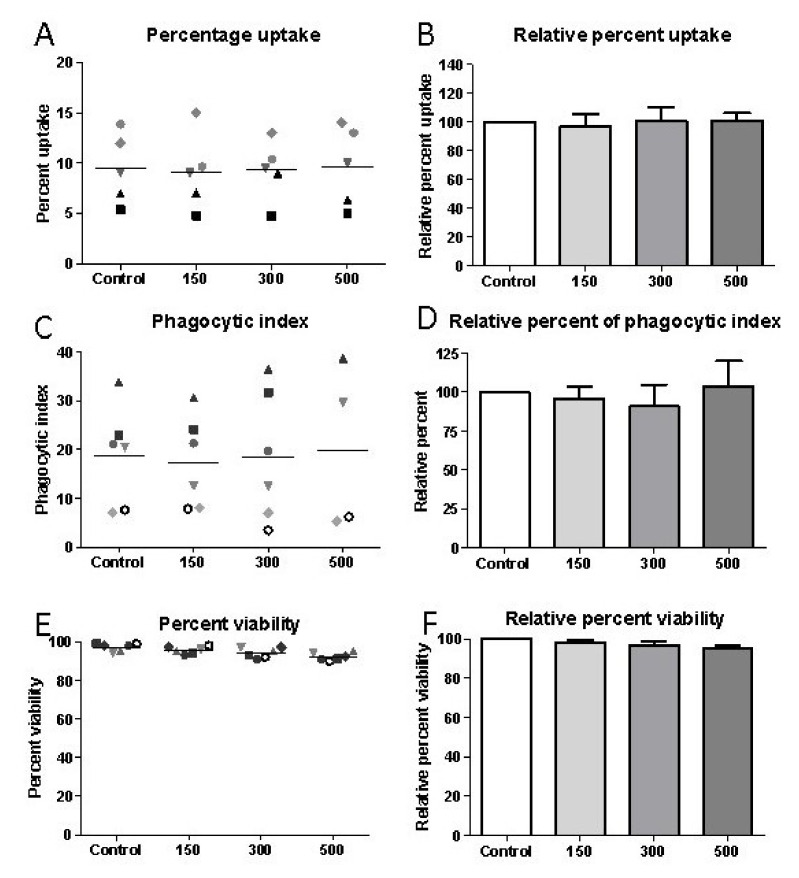
Shockwave stimulation does not up-regulate macrophage phagocytosis of the non-specific substrate, polystyrene beads. Macrophages were exposed to shockwave intensities of 150–500; 5 Hz, 0.1 mJ/mm^2^ then cultured with polystyrene beads. (**A**) Percentage uptake and (**B**) relative percentage uptake over control macrophages without shockwave exposure of polystyrene beads by J774 macrophages. (**C**) Phagocytic index and (**D**) relative percent phagocytic index over control J774 macrophages without shockwave exposure for uptake of polystyrene beads. Data plotted represent individual values as illustrated by the individual symbols (**A**,**C**) and the normalized data determining the relative percentage of shockwave exposed cells to non-exposed control cells for each cell preparation (**B**,**D**). (**E**) Percentage of viable J774 macrophages where symbols represent individual values from different experiments and (**F**) the normalized values where non-exposed control represents 100%. Data shown as means ± SEM of independent experiments from 4–7 individual macrophage preparations.

To determine if the increase in macrophage uptake by shockwaves was specific to apoptotic cells, the next experiment determined whether there were also changes in uptake of the nonspecific substrate of polystyrene beads. The percent phagocytosis was similar for the control group (9.46 ± 1.57%), and those macrophages exposed to shockwaves of intensity 150 pulses (9.1 ± 1.7%), 300 pulses (9.32 ± 1.34%), or 500 pulses (9.68 ± 1.77%); Figure 5A,B. There was no significant difference in terms of phagocytic index between the control (18.81 ± 4.14), 150 pulses (17.41 ± 3.82), 300 pulses (18.46 ± 5.45), or 500 pulses (19.93 ± 8.42) Figure 5C,D. This demonstrates that the increase in uptake of apoptotic cells by shockwaves is most likely influenced by explicit pathways and shockwaves do not affect the rate of uptake of a non-specific substrate such as polystyrene beads. Next, whether the effects on uptake of substrates by macrophages after exposure to shockwaves, could relate to changes in their overall viability status following treatment, was determined. For J774 macrophages, the majority of cells were alive and there was no significant change in percent viability as compared to the control group (mean ± SEM: 97.17 ± 0.87%) for all shockwave intensities; Figure 5E (95.5 ± 0.76%, 94.17 ± 1.05% and 92.17 ± 0.79%, for 150, 300 and 500 impulses, respectively). For data normalized to the control (100%), there was no significant difference between the control and the 150 impulse group; Figure 5F (98.32 ± 1.18%), the 300 impulse group (96.98 ± 1.78%), or the 500 impulse group (94.93 ± 1.65%). For human macrophages, there was no significant decrease in percent viability between the control group (98.75 ± 0.75%), 150 shock group (98.75 ± 1.25%), or 300 shock group (97.25 ± 1.25%). These data demonstrate that shockwave application does not significantly impact macrophage viability at the low intensity, clinically relevant treatment levels of shockwave tested.

### 2.5. Shockwave Treatment Enhances Macrophage Expression of Cytokines and Growth Factors

**Figure 6:**
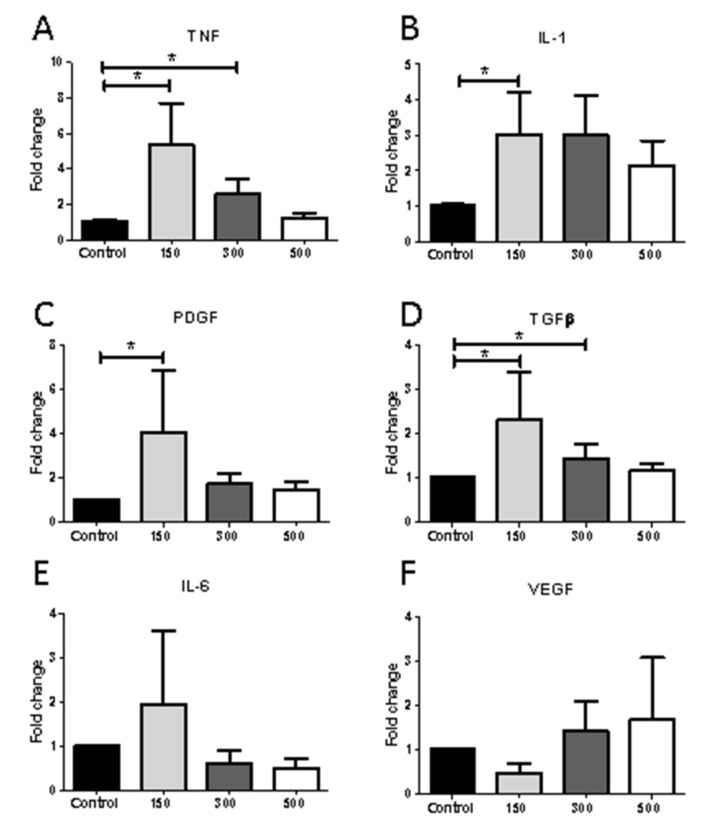
Shockwave stimulation selectively enhances macrophage mRNA expression. J774 macrophages were exposed to shockwave intensities of 150–500; 5 Hz, 0.1 mJ/mm^2^ and after 4 h, macrophage gene expression levels of (**A**) TNF, (**B**) IL-1, (**C**) PDGF, (**D**) TGFβ, (**E**) IL-6 and (**F**) VEGF, were analyzed by qPCR. Results were normalized on the housekeeping gene and expressed as fold enrichment compared to untreated control macrophages (control). Results are shown as mean ± SEM of 3–5 independent experiments, * *p* < 0.05.

Macrophages induce healing through activation-induced secretion of mediators such as cytokines and growth factors. To determine if shockwave treatment resulted in a change of mediators influencing the healing process, macrophages were subject to different intensities of shockwave and gene expression levels determined by qPCR. There was a significant increase in expression of TNF (*p* = 0.036), IL-1 (*p* = 0.029), PDGF (*p* = 0.029) and TGFβ (*p* = 0.008) at intensity of 150 and TNF (*p* = 0.034) and TGFβ (*p* = 0.048) also showed significantly increased expression at an intensity of 300 impulses (Figure 6). This suggests an activation of macrophages by shockwave treatment, consistent with the trend suggesting macrophage activation observed in human biopsies following shockwave treatment. No significant change in expression levels of IL-6 and VEGF were observed over the time periods and conditions examined.

**Figure 7:**
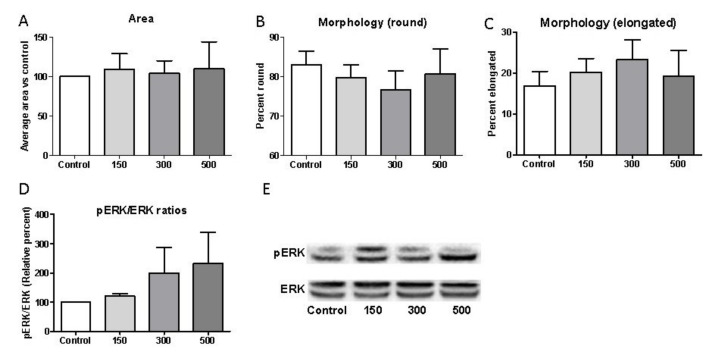
Shockwave treatment alters macrophage morphology and activates ERK signaling. J774 macrophages were exposed to shockwave intensities of 150–500; 5 Hz, 0.1 mJ/mm^2^ or left unstimulated (control). (**A**) Cell area and (**B**) changes in the percentage of J774 macrophages with rounded morphology and (**C**) elongated morphology as determined by aspect ratios; mean values ± SEM; *n* = 4 independent experiments; average 100 cells analyzed per preparation. (**D**) Shockwave stimulation induces ERK phosphorylation. (**E**) Representative Western blot for pERK and total ERK 30 min post-shockwave stimulation. Quiescent J774 cells were stimulated with shockwaves for 30 min. Total cell lysates were subjected to SDS-PAGE. Phospho-ERK mAbs were used to detect the active form of the kinase. The same blots were re-probed with antibodies against total ERK as loading controls. Quantification of protein phosphorylation levels was performed by scanning densitometry and presented as percentage increase above control non-exposed levels (mean ± SEM, *n* = 3).

We extended our finding that shockwave treatment enhances macrophage activation by defining whether exposure could influence cell morphology as a measure of activation [21,22,23]. Images of fixed J774 macrophages representing 1-h post-shockwave treatment were analyzed morphologically by comparing total cell area and by classifying cells as either elongated or rounded where the degree of cell elongation was defined as the length of the longest axis compared to the length of the short axis across the cell nucleus. The mean cell area of 100 cells was normalized to control, non-exposed cells for each set of images. There was a minor but non-significant increase in area with shockwave treatment when comparing control, 150 impulse (109.1 ± 19.96%), 300 impulse (104.1 ± 15.81%), and 500 impulse images (109.5 ± 34.48%) (Figure 7A). The mean percentage of macrophages that exhibited a rounded morphology appeared to be marginally less following application of shockwave treatment at 150 impulses than the percentage that were not exposed to shockwaves (Figure 7B). However, this decrease did not reach statistical significance; control (mean ± SEM: 83.08 ± 3.46%), 150 impulse group (79.79 ± 3.33%), 300 impulse group (76.71 ± 4.81%), and 500 impulse group (80.77 ± 6.31%). This decrease in the percentage of cells with rounded morphology was reflected in the increase of elongated cells (Figure 7C); control (16.93 ± 3.46%), 150 impulse (20.22 ± 3.33%), 300 impulse (23.30 ± 4.81%), and 500 impulse groups (19.23 ± 6.31%). As elongated cells are considered to represent an activated phenotype these findings corroborate the previous results suggesting an increase in activation by shockwave treatment; however, the effects on activation are minimal with a much greater role of shockwave treatment in increasing phagocytic efficacy of apoptotic cells.

### 2.6. Shockwave Stimulation Activates ERK Signaling

Uptake of apoptotic cells and macrophage activation have been shown to involve the activation of ERK and AKT signaling, pathways that play a role in mechanotransduction via shockwave treatment in other cell types [24]. To determine if shockwave treatment in our system could also activate ERK in macrophages, the effects were determined by Western blotting. Analysis revealed that the phosphorylation levels of ERK increased following macrophage exposure to shockwaves at 30 min post-treatment (Figure 7D,E) corroborating the role of ERK as one mediator that is relevant in the shockwave-induced signaling cascade. No changes in pAKT/total AKT ratios or pSTAT3/total STAT3 were observed at 30 min post-shockwave treatment, confirming some specificity of events (Supplementary Figure S1). The percentage change in levels of pERK/ERK compared to control was clear when comparing control to 150 impulses (121.5 ± 10.9% of control, *n* = 3), and especially 300 impulses (199.9 ± 87.2%, *n* = 3) and 500 impulses (225.1 ± 111.6%, *n* = 3). This again confirms the ability of shockwave treatment to drive responses in macrophages and signaling via ERK activation, at least in part, may relate to changes in the macrophage phagocytosis and activation observed.

## 3. Discussion

In this study, we show for the first time that low intensity shockwave treatment is an important physical stimulus that regulates macrophage functions in vivo and in vitro and this is conducive to wound healing. As established from analysis of chronic venous ulcers and biopsy data, one exposure of ESWT improves healing in the majority of patients as demonstrated by a decrease in wound area, increased angiogenesis and importantly a decrease in macrophage number per biopsy area Low intensity shockwave treatment of macrophages in vitro resulted in enhanced macrophage activation as determined by increased expressions of TNF, IL-1, PDGF, and TGFβ and the overall percentage of elongated macrophages. However, the greater effect was the significantly enhanced uptake of apoptotic cells by macrophages following shockwave treatment, an effect potentially mediated, in part, through mechanotransduction via ERK activation. Collectively, these shockwave-induced biological processes have potential as a non-invasive process to reengage macrophages in the progression of healing in chronic wounds.

Low intensity shockwaves are used clinically to accelerate healing, an effect largely ascribed to increased angiogenesis and reduced inflammation in wounded tissue [25,26] although the exact underlying cellular and molecular mechanism of how this therapy aids repair, is still poorly understood. Chronic wounds fail to heal because they are stalled in the early inflammatory stage of healing and subsequent cell proliferative and tissue remodeling phases do not readily occur [2,15]. Our demonstration that ESWT restarts wound closure in patients where biopsies show a decrease in macrophage number but not in those where the number of macrophages per biopsy area remain unchanged or increase, suggests this is an important alteration induced by ESWT to encourage the in vivo healing process. It is not clear if the shockwave-induced decrease in macrophage number per biopsy area is due to increased apoptosis and clearance or a change in their migratory potential into or out of the wound area [26]. A decrease in general leukocyte infiltration was observed in the skin wounds of diabetic rats three days post-ESWT as compared with no ESWT treatment controls [11]; however, these were experimental acute wounds, unlike our human chronic ulcers.

High levels of CD68 macrophages in the dermis and wound edges in chronic leg ulcers have previously been detected [27] yet, as in our pre-ESWT ulcer biopsies, levels of macrophage activation markers remained generally low [28], suggesting potential senescence. ESWT treatment increased activation marker expression in the majority of our post-ESWT biopsies but not explicitly discriminating between M1 and M2 activation phenotypes. This lack of a specific M1 or M1 macrophage bias is not unexpected given the heterogeneity of macrophages previously identified in chronic wounds [15].

The concept of shockwave-induced macrophage activation in vivo was followed up in macrophage cultures in vitro. Activation of macrophages is recognized through a change in cell metabolism and secretion of inflammatory mediators or a change in cell morphology [14,21,22]. Chronic wound macrophages have impaired growth factor production and dysregulated inflammatory activity [15] that prevents their healing. We demonstrate here that isolated macrophages in vitro are sensitive to shockwave exposure with significantly enhanced gene expression of wound healing growth factors and cytokines, similar to those secreted by activated wound macrophages that stimulate adjacent cells to engage in repair [29]. A previous study reported shockwaves of a comparable intensity to that used in our study did not induce activation of resting macrophages [13]. However, in their study, gene expressions of conventional M1(LPS) and M2 (IL-4) activation markers were analyzed. In our study, up-regulation of a completely different set of genes relating to healing and induced in macrophages in a complex wound environment [29] signified activation.

The significant increase in activation. as determined by gene expression in our study, may be reflected in a shockwave-induced increase (up to 20%) in the percentage of elongated cells that have been reported to be indicative of an M2 activated phenotype [21,22].

Macrophages in chronic wounds have a reduced capability to phagocytose and clear apoptotic neutrophils, contributing to the failure to heal [16,30]. A key result from our in vitro studies is that shockwave treatment significantly enhanced phagocytic uptake of apoptotic cells but not polystyrene beads. The reasons for the differences in uptake by the different substrates were not explored but potentially results from their engagement of different classes of cell surface receptors on macrophages [31]. Apoptotic cell-specific receptors including phosphatidylserine receptors, integrins, and Gas6 receptors e.g., MerTK and Axl drive apoptotic cell uptake by macrophages, while scavenger receptors are important in bead uptake. Integrins are known to be major mechanosensors and low-energy shockwaves interact with integrins [24] and activate FAK by phosphorylation, thereby triggering a series of cellular signaling including ERK1/2 activation and actin remodeling, known to be important in phagocytosis and migration [32] It is of interest that low intensity ultrasound can enhance uptake of opsonized *E. coli* that engages completely different uptake receptors from that of apoptotic cells and this is mediated through enhanced actin remodeling [33] via ERK activation. Electric fields, another physical stimulus, also up-regulates macrophage phagocytosis of apoptotic cells; however, uptake of carboxylate beads and fungal pathogens were also enhanced [34].

Regardless of the exact mechanism for shockwave-induced uptake of apoptotic cells in murine or human primary macrophages, enhancement of their phagocytic clearance would be critical for restoring tissue homeostasis, not only by preventing proinflammatory responses but also by switching on the production of anti-inflammatory, wound-resolving mediators, such as VEGF, TGF-β, and PDGF and reducing the excessive abundance of M1 macrophages found in chronic wounds [15,16]. Restoration of healing in chronic wounds requires an orchestrated interaction of cell types, extracellular matrix and cytokines. Shockwave application to endothelial cells, fibroblasts and keratinocytes has previously been shown to stimulate pro-healing cytokine secretion, morphological changes and increased cell migration of keratinocytes, proliferation of fibroblasts and a pro-angiogenic activity of endothelial cells [35]. We now add macrophages to the cohort of important cells that respond to shockwaves and produce signals that interplay and collectively induce the healing process.

Shockwave treatment activates intracellular signaling cascades in other cell types including p38, AKT, ERK1/2, FAK3, Wnt, ATP/P2X7 and PERK/ATF [24,32,36]. The effect of shockwave treatment on macrophage signaling pathways remains to be fully elucidated; however, we show ERK1/2 was activated and this process can drive actin remodeling, phagocytosis and activation processes, providing one potential pathway central to the regulation of macrophage function. Previously, stimulation of macrophages by electric fields reported an activation of ERK1/2 and PI3K (AKT) and phagocytosis and migration [34]. We did not, however, observe a change in the phosphorylation of Akt in macrophages in our study, suggesting specific effects by different physical stimuli.

Patients with chronic ulcers experience significant discomfort, risk of infection, and impaired quality of life. Treatment is still sub-optimal and the success uncertain. Targeting and correcting cellular and molecular causes of prolonged inflammation and cell senescence in chronic wounds has potential to return them to healing states. ESWT, even following one round [5], can improve the outcome in many patients as shown in the current study; however, for some patients this is not effective. Our study may help explain mechanisms underlying those non-responding patients through the inability of ESWT to favorably change macrophage abundance and activity crucial in the wound healing process.

In conclusion, our findings shed new light on the underlying mechanisms by which ESWT can potentially exert its clinical effects, namely by modulating macrophage behavior to initiate wound healing processes. This suggests shockwave therapy, as an easily administered treatment with few side effects, could also be exploited as an adjuvant therapy for other macrophage-mediated disorders.

## 4. Materials and Methods

### 4.1. Patient Recruitment

Ten patient volunteers from Aberdeen Royal Infirmary Vascular Outpatient’s Department were recruited to the study after approval was granted by the National Research Ethics Service, NOSRES Committee, reference number 16/NS/0062 and screening for eligibility by the care team. All eligible patients referred to the vascular department with treatment-resistant venous ulceration and selected to undergo ESWT were given the opportunity to participate in this study. The inclusion criteria consisted of patients with reference ulcers in excess of 1.5 cm in one axis of measurement persisting despite at least 6 weeks of treatment with 4-layer compression bandaging. The exclusion criteria included patients with ankle brachial pressure index less than 0.8, wounds being actively treated with antibiotics, patients with diabetes, suspicion of malignancy within the ulcer, acute deep vein thrombosis or neuropathy. Evaluations included clinical assessment of ulcer severity status, local blood flow perfusion, wound area size and biopsy for histopathological examination and immunohistochemical analysis. Biopsies of ulcers were collected immediately before ESWT and 2 weeks post-treatment. Of the 10 patients recruited one deteriorated and a second biopsy was not obtained so was excluded from the analysis.

### 4.2. ESWT Application to Chronic Ulcers

ESWT was performed on patients at NHS Grampian by a trained professional vascular nurse. Theshockwave source used was Dermagold100 and an OP155 applicator (Tissue Regeneration Technologies, LLC, manufactured by MTS Europe GmbH, Baden-Württemberg, Germany). The treatment dosage was dependent on the ulcer size; the number of impulses equaled the treatment area in cm^2^ × 8, but at least 500 shocks at 4 Hz (equivalent to 0.11 mJ/mm^2^ energy flux density) were delivered fortnightly for three treatments and a further three if the wound was not entirely healed.

### 4.3. Tissue Biopsy Collection

Prior to biopsy, 1% lidocaine was injected for local anesthesia and full thickness punch biopsies 3mm in diameter and 3–4 mm in depth were taken immediately before shockwave therapy and two weeks post-treatment to assess the inflammatory response and the cellular/molecular healing parameters. Biopsies were collected from the ulcer tissue adjacent to intact peri-ulcer skin (wound margin) and were taken at opposite poles of the wound for the first and second biopsy. All biopsies were immediately fixed in formalin and processed and cut to 5 µm by the Pathology Department at Aberdeen Royal Infirmary. Sections were stained, with H&E for histological analysis or Masson’s trichrome for analysis of collagen fibers, by the NHS Pathology Department. Wound healing from pre- and post-shockwave biopsies was assessed and scored histologically in H&E stained sections by a consultant histopathologist, Dr E Husain, NHS Grampian in a blinded fashion by the state of the epidermis, presence of fibrin, inflammation and hemosiderin, scarring/fibrosis, capillary proliferation and edema presence. Before ESWT, samples showed signs of chronic inflammation including intracorneal neutrophils, epidermal exocytosis, spongiosis, dermal edema, vascular ectasia, and a predominantly lymphocytic and neutrophilic dermal inflammatory cell infiltrate.

### 4.4. Immunohistochemistry

Sections were deparaffinized, microwaved in citrate buffer (pH 6.0) and endogenous peroxidase activity quenched by incubation with 3% H_2_O_2_. Macrophages were detected by anti-CD68 antibodies (Agilent Technologies. Cheshire, UK M0814, clone KP1) and the DAKO Envision Detection Kit K5007 [19]. Angiogenesis and cell proliferation were determined using mouse anti-CD31, 1:1200, ab9498 Abcam, Cambridge UK), anti-SMC (clone1A4, ab7817 Abcam) and anti Ki67 (Ab16667) followed by a reaction with biotinylated secondary antibody and streptavidin–biotin–peroxidase complex. 3,3-diaminobenzidine (DAB) was used as a chromogen, and hematoxylin solution as a counterstain and nuclei blued with Scots tap water. The DAKO G2-Double staining kit, K5361 (DakoCytomation) was used for staining with anti-CD68 (as above) and the M1-macrophage markers anti HLA DR (TAI.1B5) and SOCS3 (AB16030, Abcam) and the M2-macrophage marker, anti CD163 (EDHu-1, AbD Serotec, Kidlington, UK), according to the manufacturer’s instructions. Positive staining for activation markers was detected using diaminobenzidine/Liquid Permanent-Red (DakoCytomation) [19] as shown in Supplementary Figure S2. A negative control where the primary antibody was replaced by a non-specific IgG equivalent was included in all analyses.

### 4.5. Morphometric Measurements

Entire stained sections were photographed using the slide scanner (Zeiss Axioscan Z1, Zeiss, Cambridge, UK) 20× magnification and biopsy tissue areas were calculated using Zeiss ZEN Blue software. Intact CD68-positive cells were counted and expressed as the number of positive cells /biopsy area (mm^2^). Counts were repeated until three consecutive values were obtained with a margin of error of ±5%. Both the intra-observer and inter-observer variations were found to be less than 5% (*p* < 0.002). The number of double-positive cells was counted and expressed as a percentage of total macrophages. For CD31, SMC actin and Ki67 quantification, sections were imaged using a slide scanner and analyzed using either semiautomated computerized ImageJ software or Zeiss ZEN Blue software. The stained area was divided by the total biopsy area to obtain a relative value. For the Masson’s trichrome stained sections, the mean blue intensity measurement was taken as a relative measurement for the presence of stained collagen. To normalize the data sets and obtain values relative to the pre-treatment values, the post-treatment value in each set was expressed as a percentage.

### 4.6. Cell Culture

The murine macrophage cell line J774A.1 (ECACC 91051511) and Jurkat cell line (ATCC TIB-152, clone E6-1) was cultured in DMEM and RPMI 1640 (Lonza 12-167F) respectively, supplemented or with 10% FBS, 1% penicillin/streptomycin, and 1% L-glutamine. Passage number was kept below 20 to ensure cell line integrity. Human monocyte-derived macrophages were isolated from blood of healthy adult-consenting donors, as approved by the Ethics Review Board of the College of Life Science & Medicine, University of Aberdeen. Peripheral blood mononuclear cells (PBMCs) were fractionated by density gradient centrifugation. Monocytes were isolated from PBMCs by positive selection using human CD14 microbeads to ensure a highly purified population (Miltenyi Biotec) and differentiated into macrophages over 7 d in DMEM (Lonza, Basel, Switzerland), supplemented with 1% L-glutamine, 2% penicillin/streptomycin (GE Healthcare Life Sciences, Buckinghamshire, United Kingdom) and 10% human AB+ serum [34].

### 4.7. Shockwave Application to Cultured Macrophages

Shockwave treatment was performed on macrophages in T25 or T12.5 culture flasks or Ibidi µ-slides using electrohydraulic-generated shockwaves via the DermaGold 100 (Tissue Regeneration Technologies) at the parameters of 150–500 impulses, 5Hz, and 0.1 mJ/mm^2^. To allow the unhampered physical propagation and reproducible application of shockwaves to the sample in vitro, shockwave treatment was performed using a water bath setup under uniform treatment conditions in terms of temperature and distance to the shockwave applicator, as was previously optimized [20]. This most closely mimicked the in vivo application [20]. After treatment, the number of viable cells was determined using the trypan blue exclusion method.

### 4.8. Phagocytosis Assay

Phagocytosis assays of polystyrene beads or apoptotic Jurkat cells (immortalized line of human T lymphocyte cells) were performed using the J774 mouse macrophage cell line or human monocyte-derived macrophages, with or without exposure to shockwave treatment (150–500 impulses, 5 Hz, 0.1 mJ/mm^2^). Polystyrene beads, 6 µm (Polysciences, Eppelheim, Germany) were used as simplified non-specific targets. Apoptotic Jurkat cells were obtained by exposure to UV light, which typically resulted in >70% apoptotic cells, as determined by Annexin V and propidium iodide staining. Apoptotic cells were labeled using CellTrace Far Red (ThermoFisher, Paisley, UK) as recommended by the manufacturer. For uptake assays, apoptotic cells were added to macrophages at a ratio of 10:1 and polystyrene beads (Polysciences, Eppelheim, Germany) were added to macrophages at a ratio of 3:1 [34]. At the end of the incubation period, typically 2 h after bead/apoptotic cell addition, non-engulfed cells were removed by washing. Macrophages were fixed in 4% paraformaldehyde and the percentage of macrophages phagocytosing at least 1 bead/apoptotic cell (red fluorescence) was determined by imaging using a Zeiss Axio Observer Z1 inverted microscope (20× objective) with an MRm camera for fluorescence (Brightfield and Far Red 633). At least 100 macrophages were designated for analysis. The phagocytic index of engulfed beads was calculated using the following formula: Phagocytic index = (macrophages phagocytosing/total macrophage number) × (total beads taken up by cells) [34].

### 4.9. Cell Area and Shape Analysis

Macrophages were fixed in 4% paraformaldehyde and cells observed using a light microscope (Zeiss) and the cell area and aspect ratio were calculated using Image J with >90 cells analyzed for each group per independent preparation from 5 random fields of view where every cell in the field of view was considered [34]. Representation of F-actin and nuclear staining images of elongated and round cell morphologies are shown in Supplementary Figure S3.

### 4.10. RNA Extraction and Quantitative RT-PCR

Total cellular RNA was isolated from untreated or cytokine-stimulated human monocyte-derived macrophages using Trizol extraction reagent (Thermo Fisher Scientific Life Sciences), followed by RNA clean-up using an RNeasy Mini Kit (Qiagen, Germantown, MD, USA), according to the manufacturer’s instructions. A total of 5 μg from each sample was reverse transcribed using the First Strand cDNA Synthesis Kit and Oligo(dT) 15 Primer (Promega, Southampton, United Kingdom) and SuperScript II (Thermo Fisher Scientific Life Sciences), as recommended by the manufacturers. For each gene, the PCR was performed using the following primers: TNF-α forward (CTGTAGCCCACGTCGTAGC)TNF-α reverse (TTGAGATCCATGCCGTTG);IL-6 forward (gatgagtacaaaagtcctgatcca), IL-6 reverse (ctgcagccactggttctgt); PDGF forward (caacctgaacccagaccatc) PDGF reverse (tccttttccggtttttacctg); VEGF forward (aaaaacgaaagcgcaagaaa) VEGF reverse (tttctccgctctgaacaagg) TGFβ1 forward(tggagcaacatgtggaactc) TGFβ1 reverse (gtcagcagccggttacca) IL-1β Forward (agttgacggaccccaaaag) IL-1β reverse (agctggatgctctcatcagg). Quantitative real-time PCR was carried out by LightCycler 480 (Roche Diagnostics, West Sussex, United Kingdom) with the Universal Probe Library system (Roche Diagnostics). Gene expression was analyzed using the comparative threshold method with target gene mRNA levels being normalized to β-actin (Actb) or non-POU domain-containing (Nono). Data are expressed as fold-change differences in gene levels in untreated cells compared with that of those exposed to shockwave therapy.

### 4.11. Western Blotting

Total macrophage protein lysates were extracted using 1× RIPA buffer (Sigma Aldrich, St-Louis, MO, USA; R0278) containing complete protease inhibitors and Phosphostop (Thermo 1861281). Subsequently, the protein concentration was quantified by the Pierce BCA Protein Assay Kit, Thermo Fisher 10741395 and the sample was boiled at 100˚C for 10 min with a loading buffer (Invitrogen, ThermoFisher, Paisley, UK; NP007). Equal amounts of protein of 20 µg per lane were separated using SDS-PAGE (Invitrogen NuPage 4–12% Bis-Tris NP0321) and transferred onto a HybondTM-P PVDF membrane (Amersham, GE Healthcare, Buckinghamshire, UK). Following blocking with 5% nonfat dairy milk in Tris-buffered saline containing 0.05% Tween-20 (TBST) for 1 h at room temperature, the membrane was incubated with the following primary antibodies: Rabbit anti-ERK1/2 (monoclonal antibody; 1:1000; cat. no. 4695; Cell Signaling Technology, Inc, London, UK.) and rabbit anti-p-ERK1/2 (monoclonal antibody; 1:1000; cat. no. 4370; Cell Signaling Technology, Inc.) at 4°C overnight. The following morning, the membranes were washed four times with TBST for 10 min and incubated with an HRP-conjugated goat anti-rabbit IgG secondary antibody (Cell Signaling 7074; 1:1000) for 1 h. The signal was detected with a chemiluminescence kit containing hydrogen peroxide and Luminol components for Enhanced Chemiluminescence, (Thermo (Pierce) 32106). Subsequently, the bands were semi-quantified using an iBright or LiCor imaging device.

### 4.12. Statistical Analysis

Statistical analysis was performed using GraphPad Prism 5 (version 5.04) software. Data sets were tested for normal distribution using the Shapiro–Wilk test and/or the Kolmogorov–Smirnov test (with the Dallal-Wilkinson-Lillie for corrected *p*-value). In the event of normal distribution, significance was determined using a Students t-test. In the absence of normal distribution, significance was based on the Wilcoxon matched-pairs signed-ranks test or the Mann–Whitney test. All tests were performed with 95% confidence intervals. In some cases, a one-way analysis of variance (ANOVA) followed by Bonferroni post-hoc test was used. *P*-values less than 0.05 were taken as statistically significant, and values are given as the mean ± SEM.

## Data Availability

The data presented in this study are available on request from the corresponding author.

## Supplementary Materials

The following are available online at https://www.mdpi.com/article/10.3390/ijms22157844/s1, Figure S1: Shockwave exposure does not cause a consistent change in STAT3 or AKT activation, Figure S2: Double staining for macrophages and activation markers in formalin-fixed, wax embedded human wound biopsy section, Figure S3 Different morphological characterizations of J774 cells.
