# Supplementary material for: Low Intensity Shockwave Treatment Modulates Macrophage Functions Beneficial to Healing Chronic Wounds
Source: Int J Mol Sci. 2021 Jul 22;22(15):7844. doi: 10.3390/ijms22157844 (PMC8346032; doi:10.3390/ijms22157844)
Supplement: Supplementary file 1 [file ijms-22-07844-s001.zip › ijms-1289635-supplementary.pdf]

Supplementary Figures

Low intensity shockwave treatment modulates macrophage functions beneficial to healing chronic wounds.

Jason S Holsapple[1], Ben Cooper[2], Susan H Berry[1], Aleksandra Staniszewska[1], Bruce M Dickson[1], Julie A Taylor[1], Paul Bachoo[2], Heather M Wilson[1]*

[1]School of Medicine, Medical Sciences & Nutrition, University of Aberdeen, Foresterhill, Aberdeen, AB25 2ZD, UK

[2]NHS Grampian, Department of Vascular Surgery, Foresterhill Road, Aberdeen, UK, AB25 2ZN

*Corresponding author: Dr Heather M Wilson, School of Medicine, Medical Sciences & Nutrition, Institute of Medical Sciences, University of Aberdeen, Foresterhill, Aberdeen, AB25 2ZD, UK

Tel: +44 1224 437965, E-mail: h.m.wilson@abdn.ac.uk

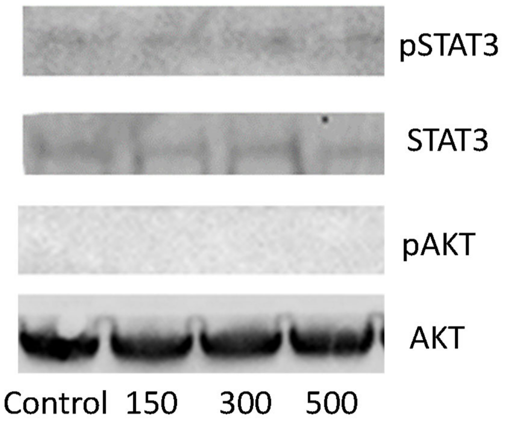

Supplementary Figure S1: Shockwave exposure does not cause a consistent change in STAT3 or AKT activation. J774 macrophage protein was isolated 30 minutes following shockwave exposure (150-500 impulses, 5Hz, 0.1 mJ/mm^2) or from non-shockwave exposed (control) cells and analysed for phospho-STAT3 (Cell Signaling Technology, Inc; Cat. No. 9131), phospho-AKT (Cell Signaling Technology, Inc; Cat. No. 9271), total STAT3 (Cell Signaling Technology, Inc; Cat. No 9132) and total AKT (Cell Signaling Technology, Inc; Cat. No 4691) by Western Blotting. The blots were stripped after probing for phosphoprotein and re-probed for total protein.

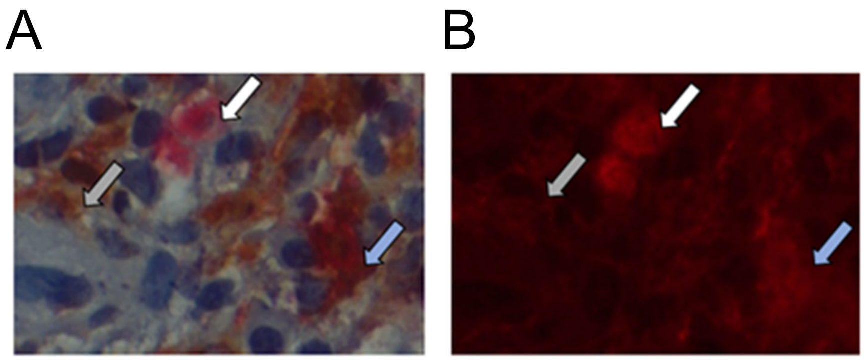

Supplementary Figure S2. Double staining for macrophages and activation markers in formalin fixed, wax embedded human wound biopsy section. Macrophages were immunostained with anti-CD68 and positive cells identified using Liquid Permanent-Red that can be detected through chromogenic red stain or fluorescence while activation markers were detected using diaminobenzidine as substrate. Fluorescent red cells were identified initially, then those also positively stained with chromogenic brown were taken as double stained cells. Picture shows cells staining singly for red (white arrow), for brown (grey arrow) and double stained for red and brown (blue arrow). (A) Stained wound biopsy under white light and (B) under fluorescence identifying single and double positive cells.

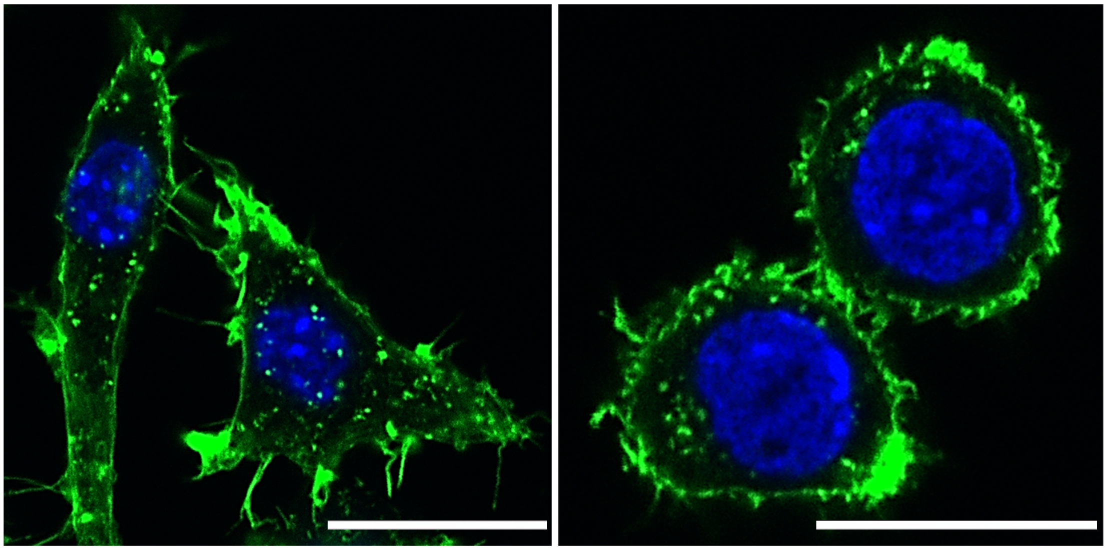

Supplementary Figure S3. Different morphological characterisations of J774 cells. Airyscan 2D-SR fluorescent images (63x objective, oil immersion) showing J774 cells; left represents an elongated phenotype while right represents rounded; Phalloidin stain detecting actin (green) and DAPI stain for nuclei (blue); scale bar = 20 μm
